# Current status of surgery first approach (part II): precautions and complications

**DOI:** 10.1186/s40902-019-0206-4

**Published:** 2019-06-03

**Authors:** Tae-Geon Kwon, Michael D. Han

**Affiliations:** 1 Department of Oral and Maxillofacial Surgery, School of Dentistry, Kyungpook National University, 2177 Dalgubeol-daero, Jung-gu, Daegu, 41940 Republic of Korea; 2 Department of Oral and Maxillofacial Surgery, College of Dentistry, University of Illinois at Chicago , Chicago, IL 60612-7211 USA

**Keywords:** Surgery first, Orthognathic surgery, Complications, Stability, Orthodontics

## Abstract

The choice of surgical technique in orthognathic surgery is based primarily on the surgical treatment objectives (STO), which is a fundamental component of the orthognathic treatment process. In the conventional orthodontics-first approach, presurgical planning can be performed twice, during the preorthodontic (initial STO) and presurgical phases (final STO). Recently, a surgery-first orthognathic approach (SFA) without presurgical orthodontic treatment has been introduced and combined initial and final STO at the same time. In contrast to the conventional surgical-orthodontic treatment protocol that includes preoperative orthodontics for dental decompensations to maximize stable postoperative occlusion, the SFA potentially shortens the treatment period and minimizes esthetic concerns during the decompensation period because skeletal problems are corrected from the beginning. The indications for the SFA have been proposed in the literature, but no consensus exists. Moreover, because dental occlusion of the pre-orthodontic arches cannot be used as a guide for establishing the surgical treatment plan, there are fundamental limitations in accurate prediction of postsurgical results in the SFA. Recently, the concepts of postsurgical orthodontic treatment are continuously changing and evolving to overcome this inherent limitation of the SFA. The elimination of presurgical orthodontics can change the paradigm of orthognathic surgery but still requires cautious case selection and thorough discussion and collaboration between orthodontists and surgeons regarding the goals and postoperative management of the orthognathic procedure.

## Background

**Fig. 1:**
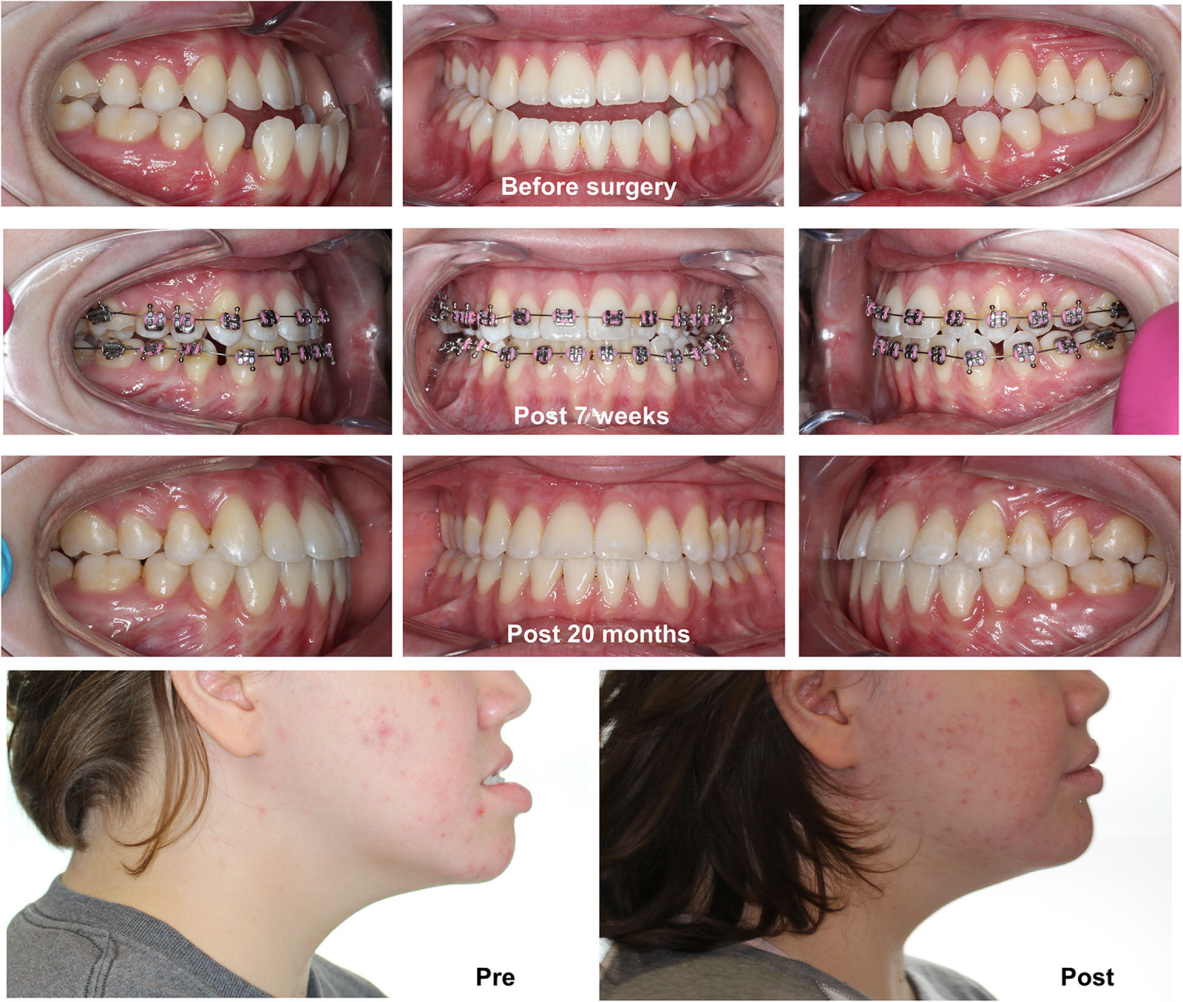
A case with surgery first approach (SFA). An 18-year-old female patient had been successfully treated by two jaw surgery followed by the 20 months of postsurgical orthodontics

The conventional orthognathic approach, or an orthodontics-first approach, involves orthodontic decompensation before surgery. This conventional approach aims to allow optimal surgical correction of skeletal deformities by defining the severity of any skeletal problems before surgery. However, skeletal deformities become more evident or even severely aggravated during the presurgical orthodontic phase, and this is a major complaint among patients, especially those with class III dentofacial deformities. The recently proposed “surgery first approach (SFA)” eliminates or minimizes such esthetic concerns during the presurgical orthodontic periods and also shortens the treatment period [1, 2]. Because of these advantages, the SFA has gained more attention in surgical-orthodontic correction of dentofacial deformities [3, 4]. Most studies on the SFA emphasize increased patient satisfaction and shorter treatment time as major advantages [2–7] (Fig. 1). Therefore, SFA is now the significant trends in orthognathic surgery.

However, previously published literature on the SFA had various methodologic shortcomings and did not utilize protocols based on scientific evidence or consensus from practitioners [8]. Therefore, it is difficult to compare many of the previous reports in parallel. In a literature review, Peiro-Guijarro et al. [9] identified 11 articles out of 179 published articles on the SFA from 2000 to 2015 that met meta-analysis guidelines. Yang et al. [10] could identify only 10 scientific articles from 342 published articles from 2001 to 2016. The enthusiasm for this new orthognathic concept often makes the clinician overlook the importance of understanding the limitations and complication of the SFA [11]. The purpose of this review was to discuss the frequently raised questions on the SFA regarding case selection criteria and potential complications and to suggest recommendations for treatment based on the existing literature and the authors’ experience.

## Review

The definition of SFA is usually “orthognathic surgery without presurgical orthodontics” [12]. However, there is a wide range of definitions concerning the “presurgical orthodontics.” For convenience and shortening the treatment time, orthodontic brackets are frequently positioned with light round or rectangular wires before surgery even in the SFA. The several weeks from bracket positioning to orthognathic surgery can be defined as a preparatory step for surgery instead of as a presurgical orthodontic period. The term “minimal presurgical orthodontics” has been frequently used as alternative terms for the SFA [13–17], but sometimes, this minimum presurgical orthodontics can potentially include the so-called early surgery approach that involves very brief presurgical orthodontics [12]. In this review, the term “minimal presurgical orthodontics” will not be used in order to clarify the true meaning and concept of the SFA.

### Fundamental questions concerning the SFA

There are definitive advantages in the SFA compared to the conventional approach. However, there are currently five fundamental questions based on the previous studies [8, 9, 11] and the authors’ experience: (1) Can the SFA ensure predictable or accurate outcomes?, (2) What is the current consensus on the indications and contraindications?, (3) Does SFA requires more number of surgeries?, (4) Can the SFA shortens the postoperative treatment period compared to the conventional approach?, (5) Does SFA shows similar stability compared to the conventional approach?

#### Is it predictable?

In the conventional orthodontics-first approach, presurgical planning can be performed twice: during the preorthodontic (initial surgical treatment objective, STO) and presurgical phases (final STO). Therefore, the surgical simulation and planning can be modified at the final STO, based on the orthodontic changes made during that interval. However, for the SFA, the STO can be performed only once (in other words, the initial STO *is* the final STO), which means that the presurgical orthodontics need to be accurately predicted, with no luxury to modify the STO based on the actual presurgical orthodontic changes as is the case in the conventional approach. Where there is arch width discrepancy, asymmetric transverse arch, or severe crossbite or deep bite, it is difficult to simulate the possible orthodontic movements that can address these problems. Therefore, accurate prediction of the occlusion after post-orthodontic treatment in the SFA is extremely challenging in such cases. Even though three-dimensional (3D) virtual occlusal setups have been utilized, it is difficult to accurately predict and simulate the occlusion after postoperative orthodontic treatment. Frequently, multi-segment osteotomies are planned in the SFA instead of simulating postsurgical orthodontic movements [18, 19]. If the surgeons establish the treatment planning based on the skeleton, the indicated postoperative orthodontic movements need to be followed. A recent report claimed that computer-aided planning in the SFA is comparable to virtual planning for a conventional approach and that routine application of 3D simulation in SFA would be possible [20]. However, introduction of 3D virtual orthodontic setup technology cannot completely solve the potential problems in discrepancies between virtually planned orthodontic movements and the actual ones [9]. There is a lack of discussion on predictions regarding occlusion, and soft tissue prediction in the peri-nasal or lip areas still requires further development [21, 22].

#### Is there a consensus on the indications and contraindications?

The selection criteria for the SFA are largely dependent on the experience and preference of the surgeon and the orthodontist. There is no consensus on the indications and contraindications for the SFA [8–10, 23].

From early papers on the SFA, Baek et al. [24] claimed that the SFA is indicated when there is only little or no transverse discrepancy, no extractions involved, and at least three occlusal contact points between the arches. They also suggested that mild to moderate curve of Spee or vertical problem could also be acceptable for the SFA.

Liou et al. [19, 25] mentioned that normal to mildly proclined/retroclined incisor inclination could be permissible in the SFA. These articles implied that cases with severe proclined/retroclined incisors or vertical problems would be the contraindication for the SFA. Later, the “inferior subapical osteotomy” has been proposed to surgically decompensate the severely retroclined mandibular incisors [26]. This also illustrates the changes in the indications and contraindications with the evolution of technique and clinical experience.

In patients with facial asymmetry, it is difficult to determine whether the case can be treated or cannot be treated by SFA. If there is significant facial asymmetry or transverse arch discrepancy, the tooth also exhibits an axial inclination to the basal bone. The ultimate correction of midline deviation or occlusal discrepancy is very difficult without dental decompensation. Therefore, these cases were discouraged to be treated with SFA [27]. However, some reports had shown that SFA could be successfully applied to the cases with severe facial asymmetry [28, 29]. It is still unclear how much asymmetry or transverse discrepancy can be accepted in the SFA [27, 30].

The same is true for an open bite. Formerly, the case with anticipated open bite after surgery would be discouraged by the SFA [7, 27]. However, it had been reported that open bite could be successfully treated by the SFA [31]. Currently, the indication barriers for the SFA are continuously changing and are sometimes overcome by technological advancements.

As surgical technique improves, the only consensus on contraindications for the SFA might be any occlusal condition that could potentially compromise the surgical procedure or the clinical results, which is not clear-cut but rather exists in the gray zone.

#### Does the SFA require more surgical intervention?

**Fig. 2:**
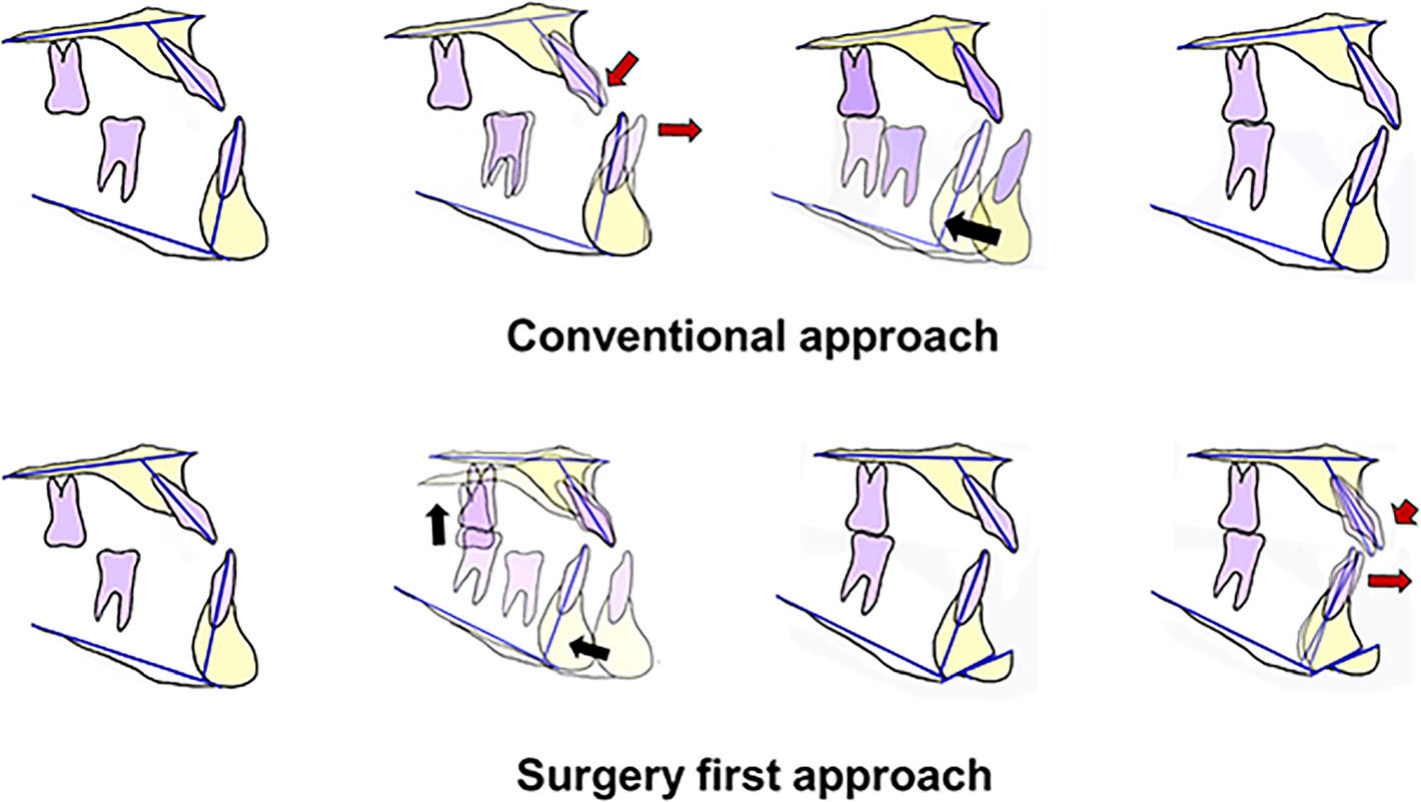
Overall concept of the SFA compared to the conventional approach. Instead of dental decompensation using the orthodontic treatment before surgery, SFA utilizes more surgical approaches for dental decompensations (red arrow, orthodontic treatment; black arrow, surgical movements)

In the abovementioned review of the literature, 84.7% of the reported SFA cases were two-jaw surgery [9]. Since the majority of the SFA has been applied to correct skeletal class III deformities, it is mandatory to correct the protruded maxillary incisor angulation by Le Fort I posterior impaction with or without segmental maxillary osteotomy [18, 32]. In the orthodontics-first approach, maxillary premolar extraction with anterior retraction can improve maxillary incisor inclination. Presurgical orthodontic treatment can be more time-consuming than the SFA but can minimize additional surgical intervention such as surgical correction of transverse discrepancies. The comparison of the SFA and conventional approaches are illustrated in Fig. 2. The recent development of screw or plate-anchored orthodontic treatment can allow minimally invasive and fewer surgical procedures [1]. Therefore, patients need to be informed that the SFA may require more surgical intervention whereas it has faster improvement of the facial profile and accelerates the treatment process.

#### Does the SFA shorten the duration of treatment and ensure stable results?

**Fig. 3:**
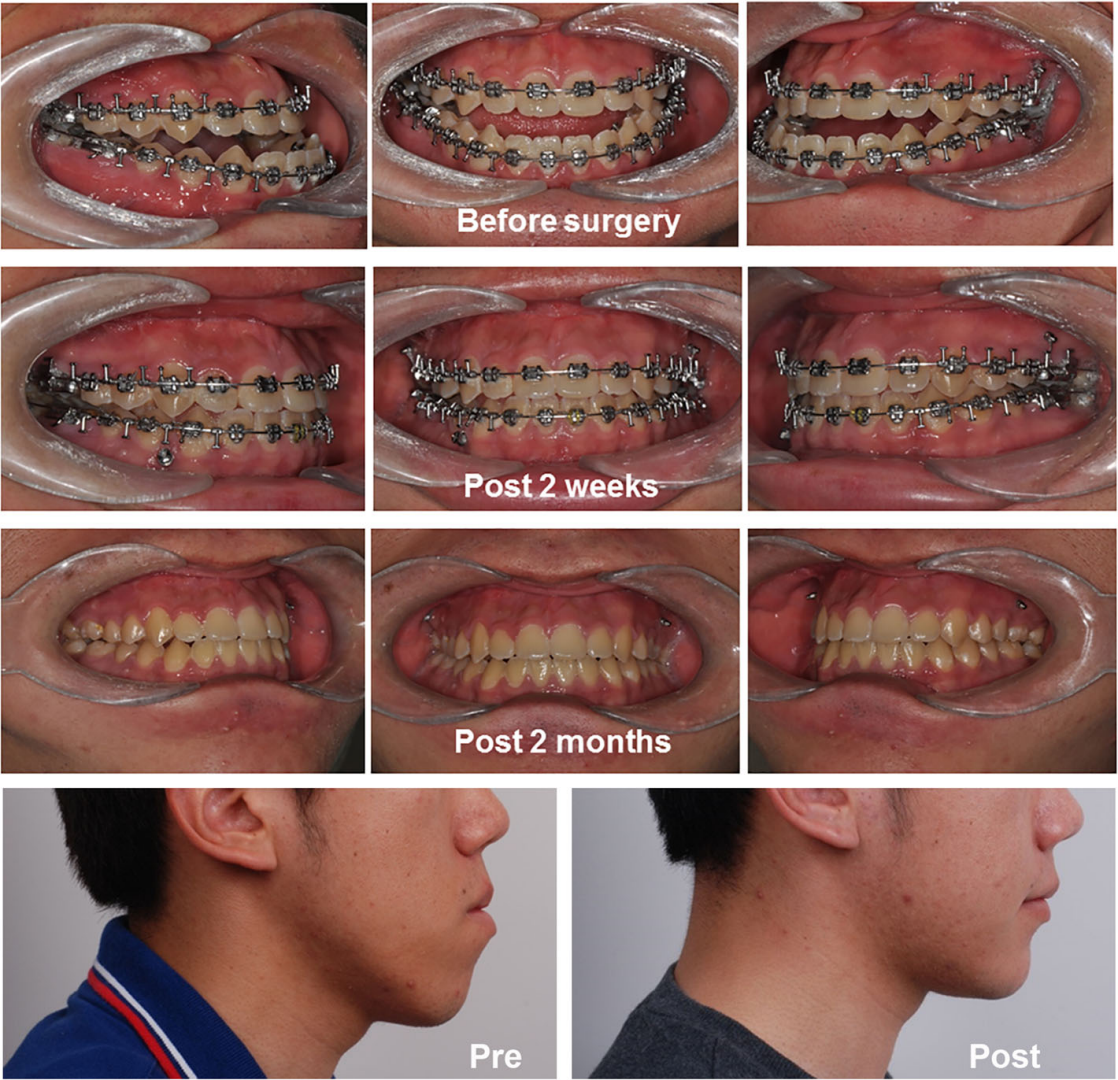
A case with the conventional approach (21-year-old male). After 4 months of presurgical orthodontics, Le Fort I osteotomy and bilateral sagittal split ramus osteotomy for mandibular setback had been performed. The overall treatment was finished after the 2 months of postoperative orthodontics

Various studies and a meta-analysis have shown that the SFA could shorten total treatment time [9, 10]. However, this does not necessarily mean that the postoperative orthodontic period in the SFA is also shorter than in the conventional approach. Rather, the postoperative orthodontic period appears to be significantly longer in the SFA compared to the conventional orthodontics-first approach [10]. This implies that orthodontic treatment in the SFA requires greater attention. However, sometimes well-planned conventional approach cases also can have shorter treatment periods (Fig. 3).

The stability of the SFA seems to be comparable to the conventional approach. Most of the meta-analysis investigations and case series on the relapse of the SFA have shown that relapse of the maxilla and mandible was not significantly higher in the SFA [10, 33, 34].

**Fig. 4:**
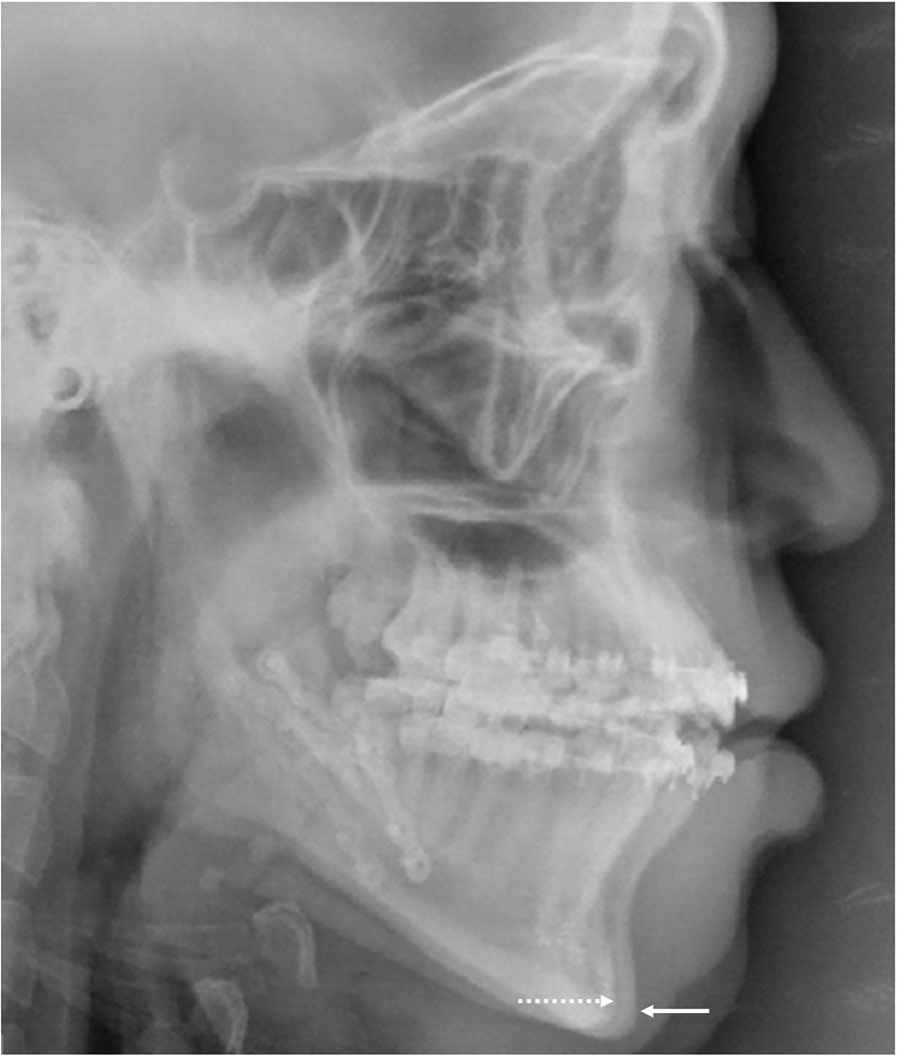
An example of mandibular relapse after SFA. Pogonion position at the immediate postoperative mark (broken arrow), significantly moved forward after surgery with SFA (solid arrow) without any evidence of temporomandibular joint problems

However, the stability issue on SFA had been continuously suggested. It had been reported that compared to the conventional approach, mandibular forward relapse was more significant in two-jaw or isolated mandibular surgery with the SFA [16, 18, 35–37]. It has been suggested that more than 50% of SFA patients with class III deformities exhibit greater than 2 mm of relapse at the pogonion [18]. Also, a greater number of higher relapse were found in the SFA group than the conventional group (57.9% versus 26.3%) [38]. SFA cases have been shown to increase vertical dimension caused by the premature contacts and to exhibit more vertical bite settling after the surgery which could result in subsequent forward mandibular relapse postoperatively [16, 18]. The authors of these studies emphasized attention to the increased the vertical dimension after orthognathic surgery with the SFA. Also, the magnitude of surgical movement would be larger than the conventional approach because of the additional amount of surgical movement of the maxilla or the mandible needed to cover the decompensatory presurgical orthodontic movements used in the conventional approach [24, 39]. Therefore, even though the stability of the SFA would be comparable to the conventional approach, possible mandibular anterior relapse must be considered during the treatment planning period (Fig. 4).

### Presurgical preparations of the SFA vs the conventional approach

It has been suggested that bone remodeling after fractures or surgical insult exceeds that seen in normal bone turnover, and this is described as “regional acceleratory phenomenon (RAP)” [40]. Liou et al. suggested that when orthognathic surgery is performed first, it can enhance the RAP. Therefore, the SFA would allow the clinician to take advantage of the RAP for orthodontic tooth movements [25]. The authors suggested the significantly elevated bone turnover marker levels from the first week to months after surgery as proof of the RAP. However, changes in biochemical markers for bone turnover from injury in extremities such the tibia, fibula, and malleolus have been shown to occur and to last 6 weeks or 6 months, and even up to a year [41, 42]. It has been already proven that this regional reaction of enhanced bone formation also can influence systemic bone metabolism. The cancellous bone compartment is greatly influenced by such systemic biological changes of the body, termed “systemic acceleratory phenomenon (SAP)” [43]. Localized bone formation after trauma or surgical insult not only leads to RAP in injured regional tissues but also induces the SAP in distant skeletal structures [44]. Therefore, accelerated tooth movement and alveolar bone remodeling during postsurgical orthodontic treatment could be attributed to both RAP and SAP.

Although the SFA does not involve presurgical orthodontics, fixed orthodontic appliances are frequently placed preoperatively to facilitate postoperative orthodontic treatment. To take advantage of locoregional or systemic acceleration of osteogenesis after surgery [25], it is important that the orthodontic brackets and archwires or mini screws are placed prior to the surgery. If these are not placed before surgery, placement in the immediate postoperative period is often very difficult for the patients because of swelling, discomfort, and limited mouth opening during this time.

**Fig. 5:**
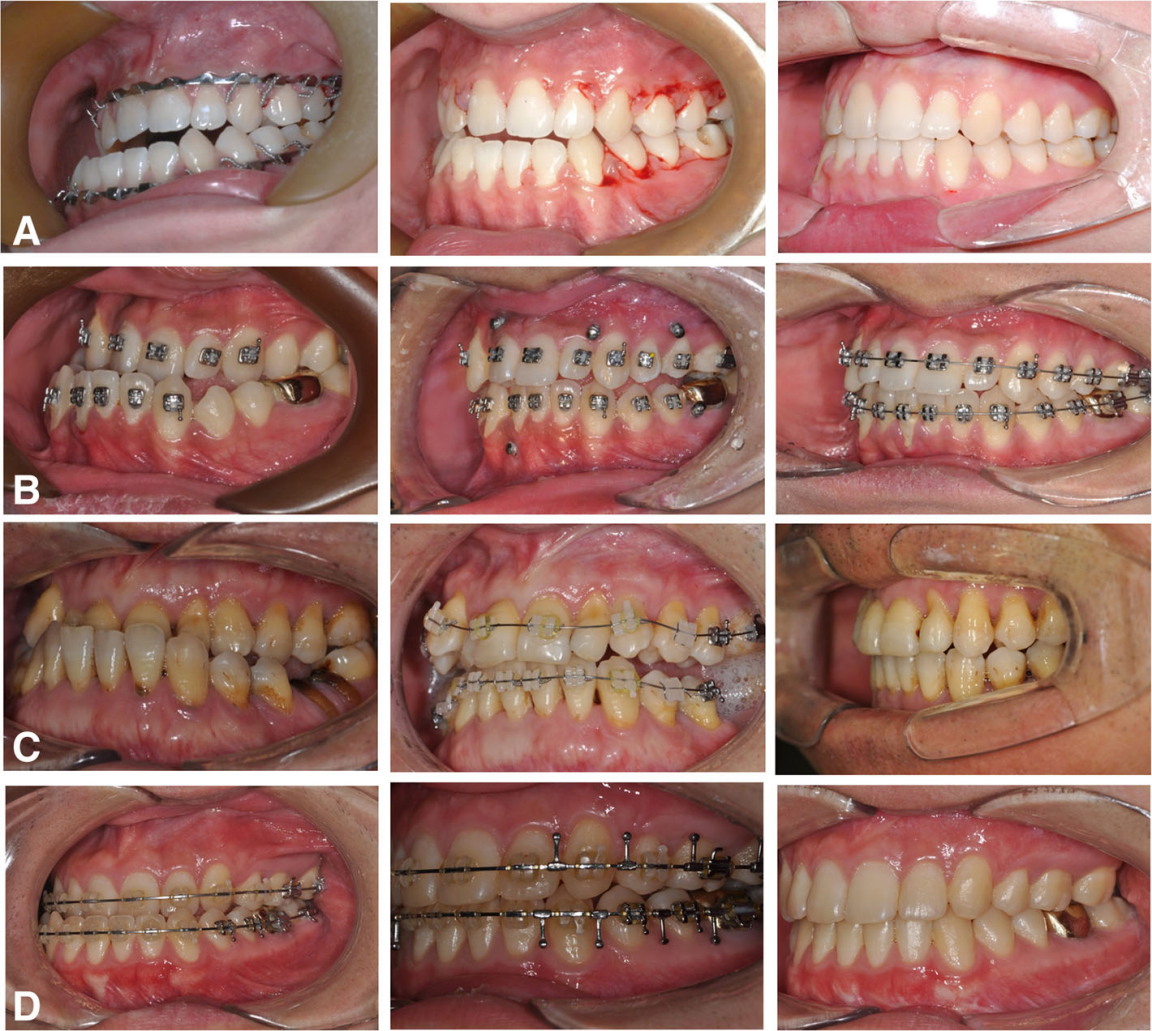
Presurgical orthodontic preparations for the SFA (**a**, **b**, **c**) versus the conventional approach (**d**). Arch bars (**a**), brackets with maxillomandibular anchor screws without archwire (**b**), or light rectangular stainless steel wires are frequently used in the SFA, whereas strong rectangular surgical wires with surgical hooks are commonly used in the conventional approach (**d**). Photos located in the left column, before surgery; middle column, immediately after surgery; right column, at the time of debond

Regarding postoperative orthodontics, there can be several options for presurgical preparation for the SFA according to the literature [1, 18, 24, 27, 37, 39, 45]: (1) preoperative placement of surgical arch bar, without orthodontic archwire, (2) preoperative placement of anchor screws, without orthodontic archwire, (3) preoperative placement of light round or light rectangular wire (with/without screws or anchor plates), (4) preoperative placement of conventional passive, rectangular wires attached with surgical hook (with/without anchor screws) (Fig. 5).

**Fig. 6:**
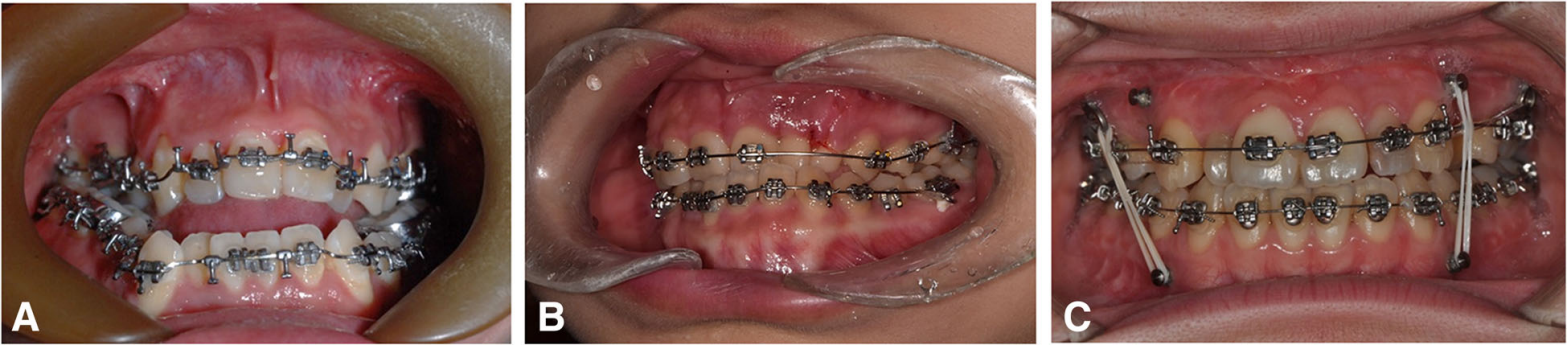
**a** For the SFA, it is difficult to passively adapt the surgical rectangular wire to the irregular dentition. **b** To maintain passivity of the surgical archwire, not all the teeth are bracketed. **c** 016 × 016 light rectangular wire with MMF screws are commonly used for surgery-first cases

**Fig. 7:**
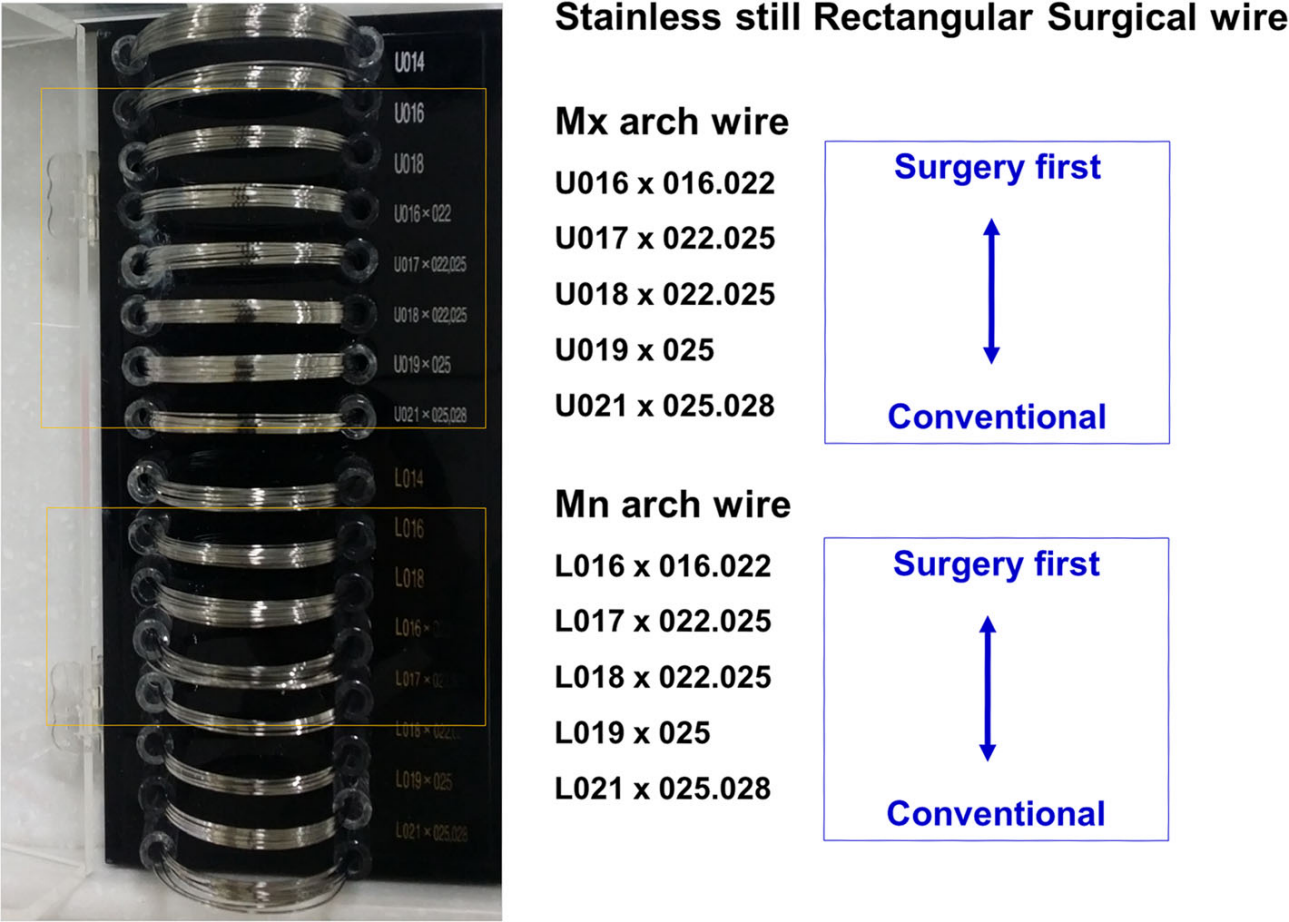
An example of suggestions for the use of surgical archwires for surgery-first and conventional approaches for orthognathic surgery

Since surgical hooks cannot be placed on light round or weak rectangular wires, additional maxillomandibular fixation (MMF) screws or anchor miniplates are frequently utilized. Passive adaptation of conventional rectangular stainless steel wires is not easy for patients with severe crowding or spacing (Fig. 6). The authors’ institution utilizes anchor screws rather than orthodontic hooks for MMF and postoperative elastic traction in the SFA. Alternatively, Kobayashi hooks or eyelet wires can be used for intraoperative MMF or postoperative guiding elastics. An example of the use of surgical arch wires for SFA and conventional approaches for orthognathic surgery are suggested in Fig. 7.

**Fig. 8:**
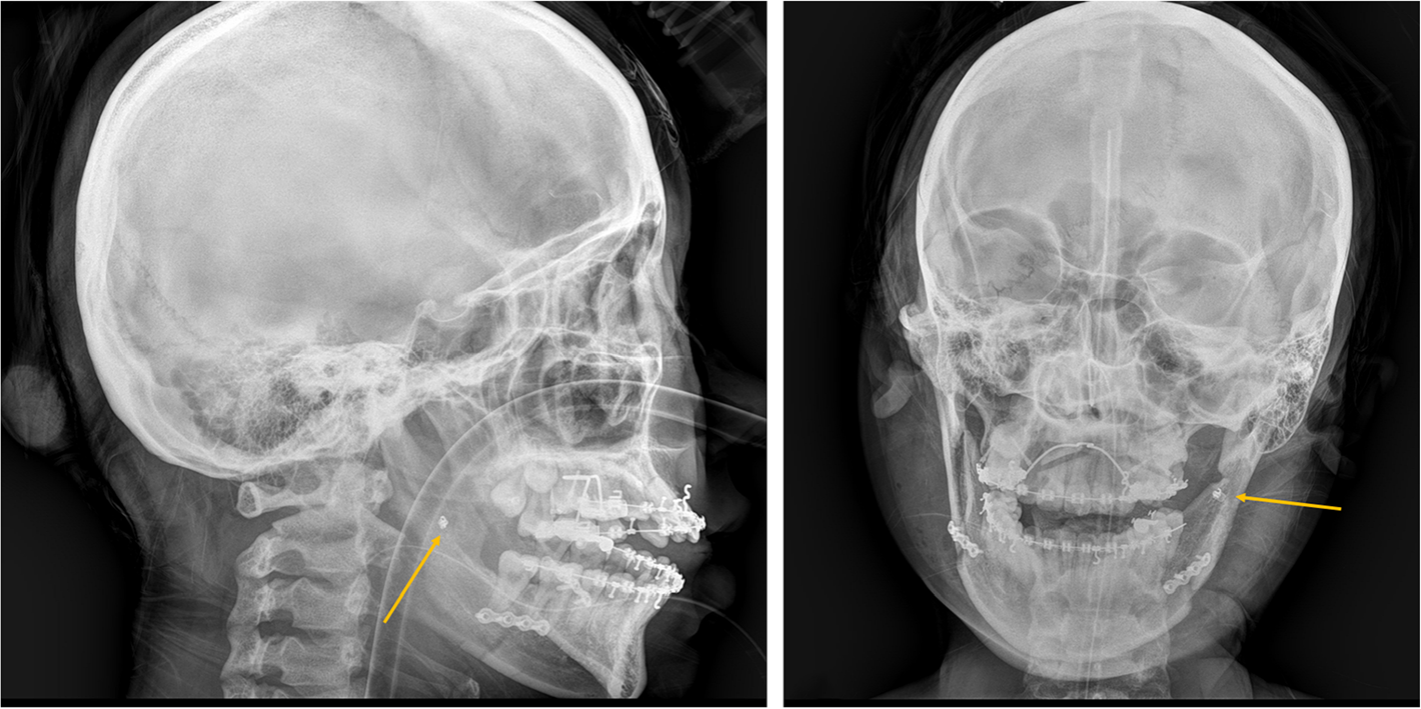
Failed brackets (arrows) are investigated in the operating room using intraoperative radiographic images (**a** lateral; **b** frontal image)

Another concern in the SFA is bracket failure during surgery [9]. Bracket failure during surgery is not infrequent and can occur in both the surgery-first and conventional approaches (Fig. 8). The incidence of bracket failure (missing or loosening) has been reported to be 16% in patients who had orthodontic brackets used for MMF during conventional orthognathic surgery. The incidence of bracket failure was higher in two-jaw surgery cases [46]. Since the SFA involves more two-jaw surgeries and orthodontic brackets are not usually placed with strong surgical archwires, the brackets and wires in the SFA frequently cannot bear or distribute the tightening stress during MMF. Therefore, the potential risk of bracket failure might be higher than that in the conventional approach. MMF screws that inadvertently placed in contact with dental roots did not cause significant risk of pulpal necrosis or pain [47]. It would be better to apply additional screws rather than relying on the brackets, especially for the SFA. The protocols for presurgical preparation for the SFA vary depending on the surgeon and the orthodontist’s preferences. When multi-segment surgery is planned, full brackets with surgical wires are strongly preferred to light wires or anchor screws. Unlike the conventional approach, it would be practical to use anchor screws for intraoperative MMF, and light round/rectangular wire can be placed for easy and fast start of postoperative orthodontics.

Where there is occlusal interference before surgery, surgeons frequently adjust occlusion by eliminating the premature contacts or high points immediately before or during orthognathic surgery. While these occlusal interferences can be minimized by presurgical orthodontics, the SFA requires more frequent reduction of occlusal interferences during the surgery in comparison.

### Postoperative management

Management of postoperative physical therapy or guidance of postoperative occlusion in the SFA requires greater attention because the occlusion is completely dependent on the surgical splint. When patients have limited, unfavorable occlusal contacts after surgery, proprioception from such occlusal contacts can induce unexpected mandibular positions from posturing. These might influence the long-term outcomes of the surgery. Wearing and adjusting the surgical splint postoperatively is an important step for a stable occlusion and long-term skeletal stability. When a significant occlusal discrepancy is anticipated after surgery, buildup of occlusal resin to stabilize the immediate postoperative occlusion should be strongly considered. Since the SFA cannot achieve optimal postoperative occlusion, the postoperative management in the surgery-first and conventional approaches are almost never the same. In the SFA, unfavorable orthodontic movements after surgery can accelerate the postoperative malocclusion more quickly [37]. The use of orthopedic traction rather than orthodontic traction is emphasized when using conventional light round orthodontic wires for surgery. The protocols for postoperative management vary by the clinical setting. Most emphasize closer and more frequent follow-up at the postoperative orthodontic phase for the SFA. Thorough follow-up with the orthodontist with continuous interactive communication is needed especially for cases utilizing the SFA [1]. A recent paper from Sugawara et al. suggested that there can be the two types of SFA: an orthodontically driven style and a surgically driven style. When the surgical treatment is utilized not only for correction of skeletal problem but also for the dental problem, it can be named as “surgery-driven style” SFA [48]. Since the final occlusion is greatly dependent on the postsurgical orthodontic treatment, the establishment of a realistic surgical goal for final orthodontic settlement is important. There is a growing concern that some misled surgeons are prone to perform orthognathic surgery at first and send the patients to orthodontists after the surgical intervention is over. The SFA should not be the synonym of the surgeon-driven approach. The limitations or morbidity of the orthodontic and surgical treatment need to be more clearly discussed between the practitioners especially for the SFA.

## Conclusion

The SFA quickly corrects skeletal deformities from the beginning and can shorten the total treatment time. Various indications for the SFA have been proposed, but there is no consensus. Moreover, because dental occlusion of the pre-orthodontic arches cannot be used as a guide for establishing the surgical treatment plan, there are fundamental limitations in accurate prediction of postsurgical results in the SFA. Recently, the concepts of postsurgical orthodontic treatment are continuously changing and evolving. The elimination of presurgical orthodontics can change the paradigm of orthognathic surgery, but the SFA requires careful case selection and thorough discussion with the orthodontist regarding the goals and postoperative management of the orthognathic surgical procedure. Therefore, the orthodontist’s decision on the orthodontic feasibility weighs heavily on the decision to plan using the SFA.

## Abbreviations

MMF: Maxillomandibular fixation
RAP: Regional acceleratory phenomenon
SAP: Systemic acceleratory phenomenon
SFA: Surgery first approach
STO: Surgical treatment objectives

## References

[1] Nagasaka H, Sugawara J, Kawamura H, Nanda R (2009). “Surgery first” skeletal Class III correction using the Skeletal Anchorage System. J Clin Orthod.

[2] Pelo S, Gasparini G, Garagiola U, Cordaro M, Di Nardo F, Staderini E (2017). Surgery-first orthognathic approach vs traditional orthognathic approach: oral health-related quality of life assessed with 2 questionnaires. Am J Orthod Dentofac Orthop.

[3] Zingler S, Hakim E, Finke D, Brunner M, Saure D, Hoffmann J (2017). Surgery-first approach in orthognathic surgery: psychological and biological aspects - a prospective cohort study. J Craniomaxillofac Surg.

[4] Uribe F, Agarwal S, Shafer D, Nanda R (2015). Increasing orthodontic and orthognathic surgery treatment efficiency with a modified surgery-first approach. Am J Orthod Dentofac Orthop.

[5] Park JK, Choi JY, Yang IH, Baek SH (2015). Patient’s satisfaction in skeletal class III cases treated with two-jaw surgery using orthognathic quality of life questionnaire: conventional three-stage method versus surgery-first approach. J Craniofac Surg.

[6] Wang J, Chen W, Ni Z, Zheng M, Liang X, Zheng Y (2017). Timing of orthognathic surgery on the changes of oral health-related quality of life in Chinese orthognathic surgery patients. Am J Orthod Dentofac Orthop.

[7] Jeong WS, Choi JW, Kim DY, Lee JY, Kwon SM (2017). Can a surgery-first orthognathic approach reduce the total treatment time?. Int J Oral Maxillofac Surg.

[8] Sharma VK, Yadav K, Tandon P (2015). An overview of surgery-first approach: recent advances in orthognathic surgery. J Orthod Sci.

[9] Peiro-Guijarro MA, Guijarro-Martinez R, Hernandez-Alfaro F (2016). Surgery first in orthognathic surgery: a systematic review of the literature. Am J Orthod Dentofac Orthop.

[10] Yang L, Xiao YD, Liang YJ, Wang X, Li JY, Liao GQ (2017). Does the surgery-first approach produce better outcomes in orthognathic surgery? A systematic review and meta-analysis. J Oral Maxillofac Surg.

[11] Jeon JH (2017). Timing of orthognathic surgery: paradigm shift by surgery-first approach?. J Korean Assoc Oral Maxillofac Surg.

[12] Hernandez-Alfaro F, Guijarro-Martinez R (2014). On a definition of the appropriate timing for surgical intervention in orthognathic surgery. Int J Oral Maxillofac Surg.

[13] Zhou Y, Zhou Y, Wang X, Li Z (2016). Minimal presurgical orthodontics for a skeletal Class III patient with mandibular asymmetry. Am J Orthod Dentofac Orthop.

[14] Choi Tae-Hyun, Kim So-Hyun, Yun Pil-Young, Kim Young-Kyun, Lee Nam-Ki (2019). Factors Related to Relapse After Mandibular Setback Surgery With Minimal Presurgical Orthodontics. Journal of Oral and Maxillofacial Surgery.

[15] Lee YS, Kim YK, Yun PY, Larson BE, Lee NK (2016). Comparison of the stability after mandibular setback with minimal orthodontics of class III patients with different facial types. J Oral Maxillofac Surg.

[16] Lee JY, Kim YK, Yun PY, Lee NK, Kim JW, Choi JH (2014). Evaluation of stability after orthognathic surgery with minimal orthodontic preparation: comparison according to 3 types of fixation. J Craniofac Surg.

[17] Kim JW, Lee NK, Yun PY, Moon SW, Kim YK (2013). Postsurgical stability after mandibular setback surgery with minimal orthodontic preparation following upper premolar extraction. J Oral Maxillofac Surg.

[18] Ko EW, Hsu SS, Hsieh HY, Wang YC, Huang CS, Chen YR (2011). Comparison of progressive cephalometric changes and postsurgical stability of skeletal Class III correction with and without presurgical orthodontic treatment. J Oral Maxillofac Surg.

[19] Liou EJ, Chen PH, Wang YC, Yu CC, Huang CS, Chen YR (2011). Surgery-first accelerated orthognathic surgery: orthodontic guidelines and setup for model surgery. J Oral Maxillofac Surg.

[20] Kim JH, Park YC, Yu HS, Kim MK, Kang SH, Choi YJ (2017). Accuracy of 3-dimensional virtual surgical simulation combined with digital teeth alignment: a pilot study. J Oral Maxillofac Surg.

[21] Holzinger D, Willinger K, Millesi G, Schicho K, Breuss E, Wagner F (2019). Changes of temporomandibular joint position after surgery first orthognathic treatment concept. Sci Rep.

[22] Baik HS, Kim SY (2010). Facial soft-tissue changes in skeletal Class III orthognathic surgery patients analyzed with 3-dimensional laser scanning. Am J Orthod Dentofac Orthop.

[23] Huang CS, Hsu SS, Chen YR (2014). Systematic review of the surgery-first approach in orthognathic surgery. Biom J.

[24] Baek SH, Ahn HW, Kwon YH, Choi JY (2010). Surgery-first approach in skeletal class III malocclusion treated with 2-jaw surgery: evaluation of surgical movement and postoperative orthodontic treatment. J Craniofac Surg.

[25] Liou EJ, Chen PH, Wang YC, Yu CC, Huang CS, Chen YR (2011). Surgery-first accelerated orthognathic surgery: postoperative rapid orthodontic tooth movement. J Oral Maxillofac Surg.

[26] Hernandez-Alfaro F, Nieto MJ, Ruiz-Magaz V, Valls-Ontanon A, Mendez-Manjon I, Guijarro-Martinez R (2017). Inferior subapical osteotomy for dentoalveolar decompensation of class III malocclusion in ‘surgery-first’ and ‘surgery-early’ orthognathic treatment. Int J Oral Maxillofac Surg.

[27] Hernandez-Alfaro F, Guijarro-Martinez R, Peiro-Guijarro MA (2014). Surgery first in orthognathic surgery: what have we learned? A comprehensive workflow based on 45 consecutive cases. J Oral Maxillofac Surg.

[28] Uribe F, Chugh VK, Janakiraman N, Feldman J, Shafer D, Nanda R (2013). Treatment of severe facial asymmetry using virtual three-dimensional planning and a “surgery first” protocol. J Clin Orthod.

[29] Watanabe Y, Sasaki R, Matsuno I, Akizuki T (2019). Surgery-first orthognathic surgery for severe facial asymmetry combined with mandibular distraction osteogenesis using a three-dimensional internal distractor. J Craniofac Surg.

[30] Jeong WS, Lee JY, Choi JW (2017). Large-scale study of long-term anteroposterior stability in a surgery-first orthognathic approach without presurgical orthodontic treatment. J Craniofac Surg.

[31] Oh JY, Park JW, Baek SH (2012). Surgery-first approach in class III open-bite. J Craniofac Surg.

[32] Huang CS, Chen YR (2015). Orthodontic principles and guidelines for the surgery-first approach to orthognathic surgery. Int J Oral Maxillofac Surg.

[33] Park KH, Sandor GK, Kim YD (2016). Skeletal stability of surgery-first bimaxillary orthognathic surgery for skeletal class III malocclusion, using standardized criteria. Int J Oral Maxillofac Surg.

[34] Soverina D, Gasparini G, Pelo S, Doneddu P, Todaro M, Boniello R et al (2019) Skeletal stability in orthognathic surgery with the surgery first approach: a systematic review. Int J Oral Maxillofac Surg. 10.1016/j.ijom.2019.01.002 2019. Jan 23 [Epub ahead of print] 10.1016/j.ijom.2019.01.002 30685226

[35] Han JJ, Jung S, Park HJ, Oh HK, Kook MS (2019). Evaluation of postoperative mandibular positional changes after mandibular setback surgery in a surgery-first approach: isolated mandibular surgery versus bimaxillary surgery. J Oral Maxillofac Surg.

[36] Lee NK, Kim YK, Yun PY, Kim JW (2013). Evaluation of post-surgical relapse after mandibular setback surgery with minimal orthodontic preparation. J Craniomaxillofac Surg.

[37] Kim CS, Lee SC, Kyung HM, Park HS, Kwon TG (2014). Stability of mandibular setback surgery with and without presurgical orthodontics. J Oral Maxillofac Surg.

[38] Park HM, Yang IH, Choi JY, Lee JH, Kim MJ, Baek SH (2015). Postsurgical relapse in class III patients treated with two-jaw surgery: conventional three-stage method versus surgery-first approach. J Craniofac Surg.

[39] Wang YC, Ko EW, Huang CS, Chen YR, Takano-Yamamoto T (2010). Comparison of transverse dimensional changes in surgical skeletal Class III patients with and without presurgical orthodontics. J Oral Maxillofac Surg.

[40] Frost HM (1983). The regional acceleratory phenomenon: a review. Henry Ford Hosp Med J.

[41] Akesson K, Kakonen SM, Josefsson PO, Karlsson MK, Obrant KJ, Pettersson K (2005). Fracture-induced changes in bone turnover: a potential confounder in the use of biochemical markers in osteoporosis. J Bone Miner Metab.

[42] Stoffel K, Engler H, Kuster M, Riesen W (2007). Changes in biochemical markers after lower limb fractures. Clin Chem.

[43] Mueller M, Schilling T, Minne HW, Ziegler R (1991). A systemic acceleratory phenomenon (SAP) accompanies the regional acceleratory phenomenon (RAP) during healing of a bone defect in the rat. J Bone Miner Res.

[44] Schilling T, Muller M, Minne HW, Ziegler R (1998). Influence of inflammation-mediated osteopenia on the regional acceleratory phenomenon and the systemic acceleratory phenomenon during healing of a bone defect in the rat. Calcif Tissue Int.

[45] Jeong TM, Kim YH, Song SI (2014). Anchor plate efficiency in postoperative orthodontic treatment following orthognathic surgery via minimal presurgical orthodontic treatment. Maxillofac Plast Reconstr Surg.

[46] Attishia R, Van Sickels JE, Cunningham LL (2015). Incidence of bracket failure during orthognathic surgery: a comparison of two techniques to establish interim maxillomandibular fixation. Oral Maxillofac Surg.

[47] Camargo IB, Van Sickels JE, Laureano Filho JR, Cunningham LL (2016). Root contact with maxillomandibular fixation screws in orthognathic surgery: incidence and consequences. Int J Oral Maxillofac Surg.

[48] Sugawara J, Nagasaka H, Yamada S, Yokota S, Takahashi T, Nanda R (2018). The application of orthodontic miniplates to Sendai surgery first. Semin Orthod.

